# Analysis-ready VCF at Biobank scale using Zarr

**DOI:** 10.1101/2024.06.11.598241

**Published:** 2024-06-12

**Authors:** Eric Czech, Timothy R. Millar, Tom White, Ben Jeffery, Alistair Miles, Sam Tallman, Rafal Wojdyla, Shadi Zabad, Jeff Hammerbacher, Jerome Kelleher

**Affiliations:** 1 Related Sciences and Lincoln, Lincoln, New Zealand; 2 The New Zealand Institute for Plant & Food Research Ltd, Lincoln, New Zealand; 3 Department of Biochemistry, School of Biomedical Sciences, University of Otago, Dunedin, New Zealand; 4 Tom White Consulting Ltd., Li Ka Shing Centre for Health Information and Discovery, University of Oxford, UK; 5 Big Data Institute, Li Ka Shing Centre for Health Information and Discovery, University of Oxford, UK; 6 Wellcome Sanger Institute, McGill University, Montreal, QC, Canada; 7 Genomics England, McGill University, Montreal, QC, Canada; 8 School of Computer Science, McGill University, Montreal, QC, Canada

**Keywords:** Variant Call Format, Zarr, Analysis ready data

## Abstract

**Background:**

Variant Call Format (VCF) is the standard file format for interchanging genetic variation data and associated quality control metrics. The usual row-wise encoding of the VCF data model (either as text or packed binary) emphasises efficient retrieval of all data for a given variant, but accessing data on a field or sample basis is inefficient. Biobank scale datasets currently available consist of hundreds of thousands of whole genomes and hundreds of terabytes of compressed VCF. Row-wise data storage is fundamentally unsuitable and a more scalable approach is needed.

**Results:**

We present the VCF Zarr specification, an encoding of the VCF data model using Zarr which makes retrieving subsets of the data much more efficient. Zarr is a cloud-native format for storing multi-dimensional data, widely used in scientific computing. We show how this format is far more efficient than standard VCF based approaches, and competitive with specialised methods for storing genotype data in terms of compression ratios and calculation performance. We demonstrate the VCF Zarr format (and the vcf2zarr conversion utility) on a subset of the Genomics England aggV2 dataset comprising 78,195 samples and 59,880,903 variants, with a 5X reduction in storage and greater than 300X reduction in CPU usage in some representative benchmarks.

**Conclusions:**

Large row-encoded VCF files are a major bottleneck for current research, and storing and processing these files incurs a substantial cost. The VCF Zarr specification, building on widely-used, open-source technologies has the potential to greatly reduce these costs, and may enable a diverse ecosystem of next-generation tools for analysing genetic variation data directly from cloud-based object stores.

## Background

Variant Call Format (VCF) is the standard format for interchanging genetic variation data, encoding information about DNA sequence polymorphisms among a set of samples with associated quality control metrics and metadata [1]. Originally defined specifically as a text file, it has been refined and standardised [2] and the underlying data-model is now deeply embedded in bioinformatics practice. Dataset sizes have grown explosively since the introduction of VCF as part of 1000 Genomes project [3], with Biobank scale initiatives such as Genomics England [4], UK Biobank [5, 6, 7, 8], and the All of Us research program [9] collecting genome sequence data for hundreds of thousands of humans. Large genetic variation datasets are also being generated for other organisms and a variety of purposes including agriculture [10, 11], conservation [12] and infectious disease surveillance [13]. VCF’s simple text-based design and widespread support [14] makes it an excellent archival format, but it is an inefficient basis for analysis. Methods that require efficient access to genotype data either require conversion to the PLINK [15, 16] or BGEN [17] formats [e.g. 18, 19, 20] or use bespoke binary formats that support the required access patterns [e.g. 21, 22, 23]. While PLINK and BGEN formats are more efficient to access than VCF, neither can accommodate the full flexibility of the VCF data model and conversion is lossy. PLINK’s approach of storing the genotype matrix in uncompressed packed-binary format provides efficient access to genotype data, but file sizes are substantially larger than the equivalent compressed VCF (see Fig 2). For example, at two bits per diploid genotype, the full genotype matrix for the GraphTyper SNP dataset in the 500K UKB WGS data [8] is 116 TiB.

Processing of Biobank scale datasets can be split into a few broad categories. The most basic analysis is quality control (QC). Variant QC is an involved and multi-faceted task [24, 25, 26], often requiring interactive, exploratory analysis and incurring substantial computation over multiple QC fields. Genotype calls are sometimes refined via statistical methods, for example by phasing [27, 28, 23, 29], and imputation [21, 30, 31, 32] creating additional dataset copies. A common task to perform is a genome wide association study (GWAS) [33]. The majority of tools for performing GWAS and related analyses require data to be in PLINK or BGEN formats [e.g 16, 20, 34, 19], and so data must be “hard-called” according to some QC criteria and exported to additional copies. Finally, variation datasets are often queried in exploratory analyses, to find regions or samples of interest for a particular study [e.g. 35].

VCF cannot support any of these workflows efficiently at the Biobank scale. The most intrinsically limiting aspect of VCF’s design is its row-wise layout of data, which means that (for example) information for a particular sample or field cannot be obtained without retrieving the entire dataset. The file-oriented paradigm is also unsuited to the realities of modern datasets, which are too large to download and often required to stay in-situ by data-access agreements. Large files are currently stored in cloud environments, where the file systems that are required by classical file-oriented tools are expensively emulated on the basic building blocks of object storage. These multiple layers of inefficiencies around processing VCF data at scale in the cloud mean that it is time-consuming and expensive, and these vast datasets are not utilised to their full potential.

To achieve this full potential we need a new generation of tools that operate directly on a primary data representation that supports efficient access across a range of applications, with native support for cloud object storage. Such a representation can be termed “analysis-ready” and “cloud-native” [36]. For the representation to be FAIR [37], it must also be *accessible*, using protocols that are “open, free, and universally implementable”. There is currently no efficient, FAIR representation of genetic variation data suitable for cloud deployments. Hail [38, 39] has become the dominant platform for quality control of large-scale variation datasets, and has been instrumental in projects such as gno-madAD [40, 26]. While Hail is built on open components from the Hadoop distributed computing ecosystem [41], the details of its MatrixTable format are not documented or intended for external reuse. Similarly, commercial solutions that have emerged to facilitate the analysis of large-scale genetic variation data are either based on proprietary [42, 43, 44, 45, 46] or single-vendor technologies [e.g. 47, 48]. The next generation of VCF analysis methods requires an open, free and transparent data representation with multiple independent implementations.

In this article, we decouple the VCF data model from its row-oriented file definition, and show how the data can be compactly stored and efficiently analysed in a cloud-native, FAIR manner. We do this by translating VCF data into Zarr format, a method of storing large-scale multidimensional data as a regular grid of compressed chunks. Zarr’s elegant simplicity and first-class support for cloud object stores have led to it gaining substantial traction across the sciences, and it is now used in multiple petabyte-scale datasets in cloud deployments (see Methods for details). We present the VCF Zarr specification that formalises this mapping, and the vcf2zarr utility to reliably convert large-scale VCFs to Zarr. We show that VCF Zarr is much more compact than VCF and is competitive with state-of-the-art file-based VCF compression tools. Moreover, we show that Zarr’s storage of data in an analysis-ready format greatly facilitates computation, with various benchmarks being substantially faster than bcftools based pipelines, and again competitive with state-of-the-art file-oriented methods. Finally, we show the utility of VCF Zarr on the Genomics England aggV2 dataset, demonstrating that common bcftools queries can be performed orders of magnitude more quickly using simple Python scripts.

## Results

### Storing genetic variation data

Although VCF is the standard format for exchanging genetic variation data, its limitations both in terms of compression and query/compute performance are well known [e.g. 49, 50, 51], and many methods have been suggested to improve on these properties. Most approaches balance compression with performance on particular types of queries, typically using a command line interface (CLI) and outputting VCF text [50, 51, 52, 53, 54, 55, 56, 57, 58, 59]. Several specialised algorithms for compressing the genotype matrix (i.e., just the genotype calls without additional VCF information) have been proposed [60, 61, 62, 63, 64, 65] most notably the Positional Burrows–Wheeler Transform (PBWT) [66]. See [67] for a review of the techniques employed in genetic data compression. The widely-used PLINK binary format stores genotypes in a packed binary representation, supporting only biallelic variants without phase information. The PLINK 2 PGEN format [68] is more general and compact than PLINK, compressing variant data using specialised algorithms [62]. Methods have also been developed which store variation data along with annotations in databases to facilitate efficient queries [e.g. 69, 70] which either limit to certain classes of variant [e.g. 71] or have storage requirements larger than uncompressed VCF [72]. The SeqArray package [73] builds on the Genomic Data Storage container format [74] to store VCF genotype data in a packed and compressed format, and is used in several downstream R packages [e.g. 75, 76].

VCF is a row-wise format in which observations and metadata for a single variant are encoded as a line of text [1]. BCF [77], the standard binary representation of VCF, is similarly row-wise, as are the majority of proposed alternative storage formats. Row-wise storage makes retrieving all information for a given record straight-forward and efficient, and works well when records are either relatively small or we typically want to analyse each record in its entirety. When we want to analyse only a subset of a record, row-wise storage can be inefficient because we will usually need to retrieve more information than required from storage. In the case of VCF (and BCF) where records are not of a fixed size and are almost always compressed in blocks, accessing any information for a set of rows means retrieving and decompressing *all* information from these rows.

The usual alternative to row-wise storage is *columnar* storage: instead of grouping together all the fields for a record, we group together all the records for a given field. Columnar storage formats such as Parquet [78] make retrieving particular columns much more efficient and can lead to substantially better compression. While columnar techniques have been successfully applied in alignment storage [e.g. 79, 80, 81], the use of columnar technologies for storing and analysing variation data have had limited success [82, 83]. Mapping VCF directly to a columnar layout, in which there is a column for the genotypes (and other per-call QC metrics) for each sample leads to a large number of columns, which can be cumbersome and cause scalability issues. Fundamentally, columnar methods are one-dimensional, storing a vector of values associated with a particular key, whereas genetic variation data is usually modelled as a two-dimensional matrix in which we are interested in accessing both rows *and* columns. Just as row-oriented storage makes accessing data for a given sample inefficient, columnar storage makes accessing all the data for a given variant inefficient.

**Figure 1.**
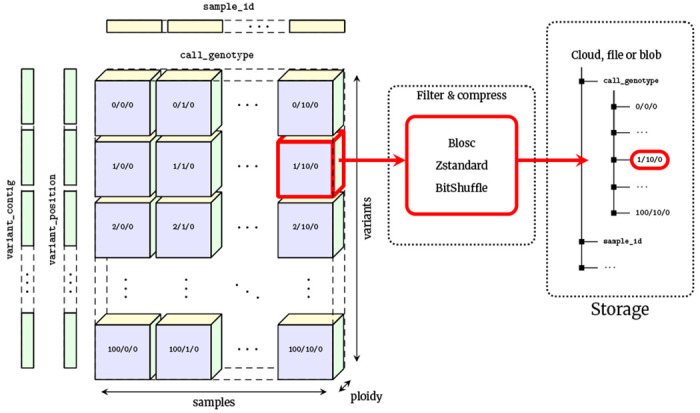
Chunked compressed storage of VCF data using Zarr. The call_genotype array is a three-dimensional (variants, samples, ploidy) array of integers, split into a uniform grid of chunks determined by the variant and sample chunk sizes (10,000 and 1,000 by default in vcf2zarr). Each chunk is associated with a key defining its location in this grid, which can be stored in any key-value store such as a standard file-system or cloud object store. Chunks are compressed independently using standard codecs and pre-compression filters, which can be specified on a per-array basis. Also shown are the one-dimensional variant_contig (CHROM) and variant_position arrays (POS). Other fields are stored in a similar fashion.

VCF is at its core an encoding of the genotype matrix, where each entry describes the observed genotypes for a given sample at a given variant site, interleaved with per-variant information and other call-level matrices (e.g., the GQ or AD fields). The data is largely numerical and of fixed dimension, and is therefore a natural mapping to array-oriented or “tensor” storage. We propose the VCF Zarr specification which maps the VCF data model into an array-oriented layout using Zarr (Fig 1). In the VCF Zarr specification, each field in a VCF is mapped to a separately-stored array, allowing for efficient retrieval and high levels of compression. See the Methods for more detail on Zarr and the VCF Zarr specification.

One of the key benefits of Zarr is its cloud-native design, but it also works well on standard file systems, where arrays and chunks are stored hierarchically in directories and files (storage as a single Zip archive is also supported). To enable comparison with the existing file-based ecosystem of tools, we focus on Zarr’s file system chunk storage in a series of illustrative benchmarks in the following sections. (See [84, 85, 86] for Zarr benchmarks in cloud settings.) We compare primarily with VCF/BCF based workflows using bcf tools because this is the standard practice, used in the vast majority of cases. We also compare with two representative recent specialised utilities; see [53, 59] for further benchmarks of these and other tools. Genozip [55, 56] is a tool focused on compression performance, which uses a custom file format and a CLI to extract VCF as text with various filtering options. Savvy [57] is an extension of BCF which takes advantage of sparsity in the genotype matrix as well as using PBWT-based approaches for improved compression. Savvy provides a CLI as well as a C++ API. Our benchmarks are based on genotype data from subsets of a large and highly realistic simulation of French-Canadians [87] (see Methods for details on the dataset and benchmarking methodology). Note that while simulations cannot capture all the subtleties of real data, the allele frequency and population structure patterns in this dataset have been shown to closely follow observations [87] and so it provides a reasonable and easily reproducible data point when comparing such methods. The simulations only contain genotypes without any additional high-entropy QC fields, which is unrealistic (see the Genomics England case-study for benchmarks on a large human dataset that includes many such fields). Note, however, that such minimal, genotype-only data is something of a best-case scenario for specialised genotype compression methods using row-wise storage.

**Figure 2.**
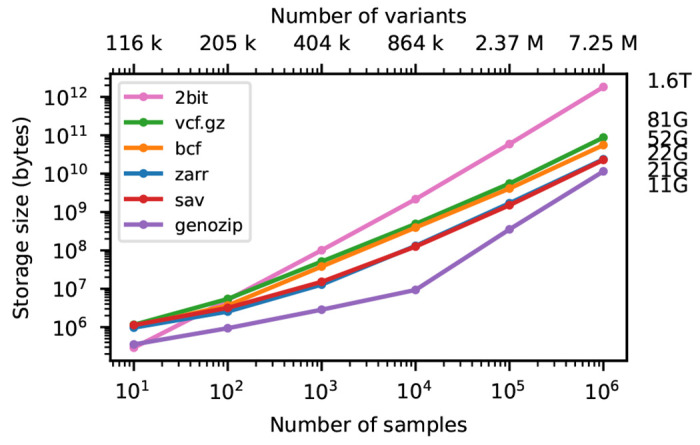
Compression performance on simulated genotypes. Comparison of total stored bytes for VCF data produced by subsets of a large simulation of French-Canadians. Sizes for 10^6^ samples are shown on the right. Sizes for Savvy (21.25GiB) and Zarr (22.06GiB) are very similar. Also shown for reference is the size of genotype matrix when encoded as two bits per diploid genotype (2bit), as used in the PLINK binary format.

Fig 2 shows compression performance on up to a million samples for chromosome 21, with the size of the genotype-matrix encoded as l-bit per haploid call included for reference. Gzip compressed VCF performs remarkably well, compressing the data to around 5% of the minimal binary encoding of a biallelic genotype matrix for 1 million samples. BCF provides a significant improvement in compression performance over VCF (note the log-log scale). Genozip has superb compression, having far smaller file sizes that the other methods (although somewhat losing its advantage at larger sample sizes). Zarr and Savvy have almost identical compression performance in this example. It is remarkable that the simple approach of compressing two dimensional chunks of the genotype matrix using the Zstandard compressor [88] and the bit-shuffle filter from Blosc [89] (see Methods for details) produces compression levels competitive with the highly specialised methods used by Savvy.

### Calculating with the genotype matrix

Storing genetic variation data compactly is important, but it is also important that we can analyse the data efficiently. Bioinformatics workflows tend to emphasise text files and command line utilities that consume and produce text [e.g. 90]. Thus, many tools that compress VCF data provide a command line utility with a query language to restrict the records examined, perform some pre-specified calculations and finally output some text, typically VCF or tab/comma separated values [50, 51, 53, 54, 55, 56, 59]. These pre-defined calculations are by necessity limited in scope, however, and the volumes of text involved in Biobank scale datasets make the classical approach of custom analyses via Unix utilities in pipelines prohibitively slow. Thus, methods have begun to provide Application Programming Interfaces (APIs), providing efficient access to genotype and other VCF data [e.g. 49, 57, 58]. By providing programmatic access, the data can be retrieved from storage, decoded and then analysed in the same memory space without additional copies and inter-process communication through pipes.

To demonstrate the accessibility of genotype data and efficiency with which calculations can be performed under the different formats, we use the bcftools +af-dist plugin (which computes a table of deviations from Hardy-Weinberg expectations in allele frequency bins) as an example. We chose this particular operation for several reasons. First, it is a straightforward calculation that requires examining every element in the genotype matrix, and can be reproduced in different programming languages without too much effort. Secondly, it produces a small volume of output and therefore the time spent outputting results is negligible. Finally, it has an efficient implementation written using the htslib C API [91], and therefore running this command on a VCF or BCF file provides a reasonable approximation of the limit of what can be achieved in terms of whole-matrix computation on these formats.

**Figure 3.**
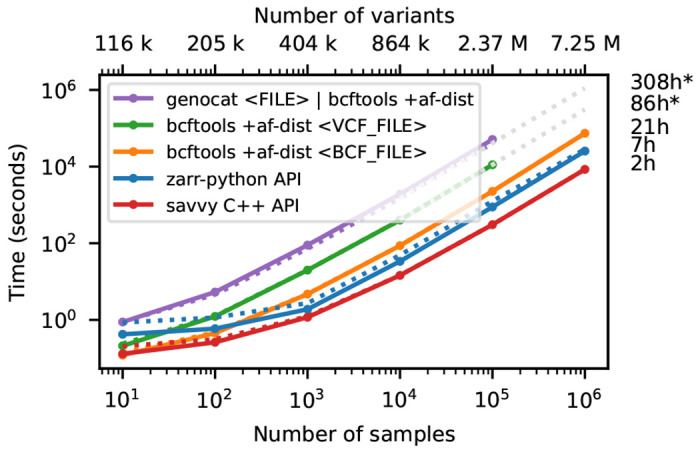
Whole-matrix compute performance with increasing sample size. Total CPU time required to run bcftools +af-dist and equivalent operations in a single thread for various tools. Elapsed time is also reported (dotted line). Run-time for genozip and bcftools on VCF at 10^6^ samples were extrapolated by fitting an exponential. See Methods for full details.

Fig 3 shows timing results for running bcftools +af-dist and equivalent operations on the data of Fig 2. There is a large difference in the time required (note the log-log scale). The slowest approach uses Genozip. Because Genozip does not provide an API and only outputs VCF text, the best approach available is to pipe its output into bcftools +af-dist. This involves first decoding the data from Genozip format, then generating large volumes of VCF text (terabytes, in the largest examples here), which we must subsequently parse before finally doing the actual calculation. Running bcftools +af-dist directly on the gzipped VCF is substantially faster, indicating that Genozip’s excellent compression performance comes at a substantial decompression cost. Using a BCF file is again significantly faster, because the packed binary format avoids the overhead of parsing VCF text into htslib’s internal data structures. We only use BCF for subsequent bcftools benchmarks.

The data shown in Fig 3 for Zarr and Savvy is based on custom programs written using their respective APIs to implement the af-dist operation. The Zarr program uses the Zarr-Python package to iterate over the decoded chunks of the genotype matrix and classifies genotypes within a chunk using a 14 line Python function, accelerated using the Numba JIT compiler [92]. The allele frequencies and genotype counts are then analysed to produce the final counts within the allele frequency bins with 9 lines of Python using NumPy [93] functions. Remarkably, this short and simple Python program is substantially faster than the equivalent compiled C using htslib APIs on BCF (6.9 hours vs 20.6 hours for 1 million samples). The fastest method is the C++ program written using the Savvy API. This would largely seem to be due to Savvy’s excellent genotype decoding performance (up to 6.6GiB/s vs 1.2GiB/s for Zarr on this dataset; Fig S1). Turning off the BitShuffle filter for the Zarr dataset, however, leads to a substantial increase in decoding speed (3.9GiB/s) at the cost of a roughly 25% increase in storage space (29.9GiB up from 22.1GiB for 1 million samples; data not shown). Given the relatively small contribution of genotypes to the overall storage of real datasets (see the Genomics England example) and the frequency that they are likely to be accessed, this would seem like a good tradeoff in most cases. This ability to easily tune compression performance and decoding speed on a field-by-field basis is a major strong point of Zarr. The vcf2zarr utility also provides functionality to aid with such storage schema tuning.

### Subsetting the genotype matrix

As datasets grow ever larger, the ability to efficiently access subsets of the data becomes increasingly important. VCF/BCF achieve efficient access to the data for genomic ranges by compressing blocks of adjacent records using bgzip, and storing secondary indexes alongside the original files with a conventional suffix [94]. Thus, for a given range query we decompress only the necessary blocks and can quickly access the required records. The row-wise nature of VCF (and most proposed alternatives), however, means that we cannot efficiently subset *by sample* (e.g., to calculate statistics within a particular cohort). In the extreme case, if we want to access only the genotypes for a single sample we must still retrieve and decompress the entire dataset.

**Figure 4.**
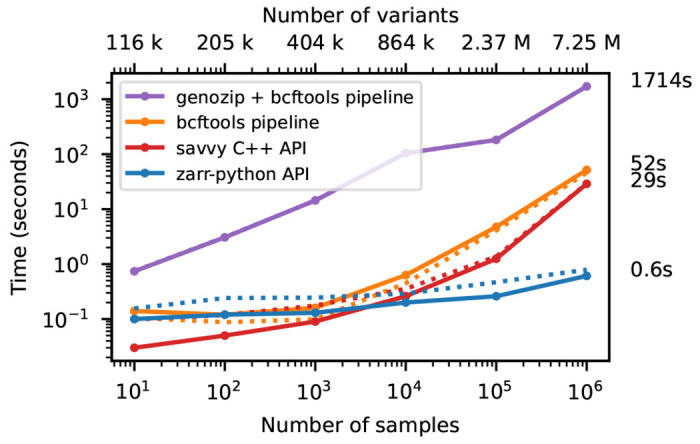
Compute performance on subsets of the matrix. Total CPU time required to run the af-dist calculation for a contiguous subset of 10,000 variants × 10 samples from the middle of the matrix for the data in Fig 2. Elapsed time is also reported (dotted line). The genozip and bcftools pipelines involve multiple commands required to correctly calculate the AF INFO field required by bcftools +af-dist. See the Methods for full details on the steps performed.

We illustrate this cost of row-wise encoding in Fig 4, where we run the af-dist calculation on a small fixed-size subset of the genotype matrices of Fig 2. The two-dimensional chunking of Zarr means that this sub-matrix can be efficiently extracted, and therefore the execution time depends very weakly on the overall dataset size, with the computation requiring around 1 second for 1 million samples. Because of their row-wise encoding, CPU time scales with the number of samples for all the other methods. Fig S2 shows performance for the same operation when selecting half of the samples in the dataset.

### Extracting, inserting and updating fields

**Figure 5.**
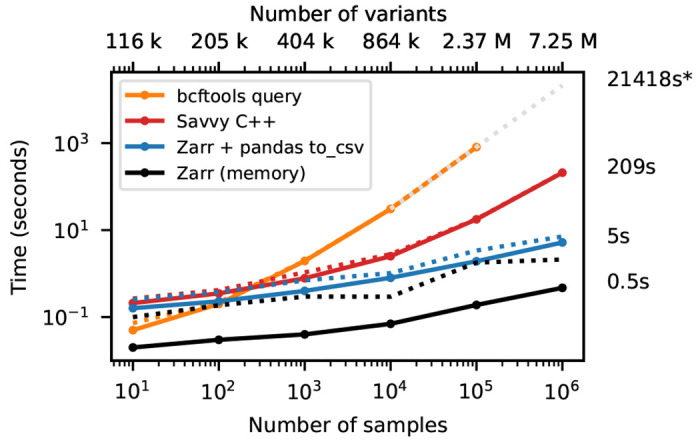
Time to extract the genome position and write to a text file. Total CPU time required to extract the POS field for BCF, sav and Zarr formats for the data in Figure 2. For the BCF file we used bcftools query -f“%POS\n”. For sav, we used the Savvy C++ API to extract position for each variant and output text using the std::cout stream. For Zarr, we read the variant_position array into a NumPy array, and then wrote to a text file using the Pandas write_csv method. Zarr CPU time is dominated by writing the text output; we also show the time required to populate a NumPy array with the data in Zarr, which is less than a second. Wall-clock time (dotted line) is dominated in this case by file I/O. Time to output text for Savvy is not significant for > 1000 samples (not shown).

We have focused on the genotype matrix up to this point, contrasting Zarr with existing row-wise methods. Real-world VCFs encapsulate much more than just the genotype matrix, and can contain large numbers of additional fields. Fig 5 shows the time required to extract the genomic position of each variant in the simulated benchmark dataset, which we can use as an indicative example of a per-variant query. Although Savvy is many times faster than bcftools query here, the row-wise storage strategy that they share means that the entire dataset must be read into memory and de-compressed to extract just one field from each record. Zarr excels at these tasks: we only read and decompress the information required.

Many of the additional fields that we find in real-world VCFs are variant-level annotations, extensively used in downstream applications. For example, a common workflow is to add or update variant IDs in a VCF using a reference database such as dbSNP [95]. The standard approach to this (using e.g. bcftools annotate) is to create a *copy* of the VCF which includes these new annotations. Thus, even though we may only be altering a single field comprising a tiny fraction of the data, we still read, decompress, update, compress and write the entire dataset to a new file. With Zarr, we can update an existing field or add arbitrary additional fields without touching the rest of the data or creating redundant copies.

### Case study: Genomics England 100,000 genomes

In this section we demonstrate the utility of VCF Zarr on a large human dataset and the scalability of the vcf2zarr conversion utility. Genomics England’s multi-sample VCF dataset (aggV2) is an aggregate of 78,195 gVCFs from rare disease and cancer participants recruited as part of the 100,000 Genomes Project [4]. The dataset comprises approximately 722 million annotated single-nucleotide variants and small indels split into 1,371 roughly equal chunks and totalling 165.3 TiB of VCF data after bgzip compression. The dataset is used for a variety of research purposes, ranging from GWAS [96] and imputation [97] to simple queries involving single gene regions [98, 99].

As described in the Methods, conversion to Zarr using vcf2zarr is a two-step process. We first converted the 106 VCF files (12.81 TiB) for chromosome 2 into the intermediate columnar format (ICF). This task was split into 14,605 partitions, and distributed using the Genomics England HPC cluster. The average run-time per partition was 20.7 min. The ICF representation used a total of 9.94 TiB over 3,960,177 data storage files. We then converted the ICF to Zarr, partitioned into 5989 independent jobs, with an 18.6 min average run time. This produced a dataset with 44 arrays, consuming a total of 2.54 TiB of storage over 6,312,488 chunk files. This is a roughly 5X reduction in total storage space over the original VCF. The top fields in terms of storage are detailed in Table 1. We do not compare with other tools such as Genozip and Savvy here because they have fundamental limitations (as shown in earlier simulation-based benchmarks), and conversion of these large VCFs is a major undertaking.

Table 1 shows that the dataset storage size is dominated by a few columns with the top four (call_AD, call_GQ, call_DP and call_DPF) accounting for 90% of the total. These fields are much less compressible than genotype data (which uses < 1% of the total space here) because of their inherent noisiness [54]. Note that these top four fields are stored as 16 bit integers because they contain rare outliers that cannot be stored as 8 bits. While the fields could likely be truncated to have a maximum of 127 with minimal loss of information, the compression gains from doing so are relatively minor, and we therefore opt for fully lossless compression here for simplicity. The call_PS field here has an extremely high compression ratio because it consists entirely of missing data (i.e., it was listed in the header but never used in the VCF).

To demonstrate the computational accessibility of Zarr on this large human dataset, we performed some illustrative benchmarks. As these benchmarks take some time to run, we focus on a single 132GiB compressed VCF file covering positions 58,219,159–60,650,943 (562,640 variants) from the middle of the list of 106 files for chromosome 2. We report both the total CPU time and elapsed wall-clock time here as both are relevant. First, we extracted the genome position for each variant in this single VCF chunk using bcftools query and Python Zarr code as described in Fig 5. The bcftools command required 55.42 min CPU and 85.85 min elapsed. The Zarr code required 2.78 sec CPU and 1.73 min elapsed. This is a 1196X smaller CPU burden and a 50X speed-up in elapsed time. The major difference between CPU time and wall-time is noteworthy here, and indicates some opportunities for improvement in VCF Zarr in high-latency environments such as the shared file system in the Genomics England HPC system. Currently VCF Zarr does not store any specialised index to map genomic coordinates to array positions along the variants dimension. Instead, to find the relevant slice of records corresponding to the range of positions in the target VCF file, we load the entire variant_position array and binary search. This entails reading 5,989 chunk files (the chunk size is 100,000 variants) which incurs a substantial latency penalty on this system. Later versions of the specification may solve this problem by storing an array of size (approximately) the number variant chunks which maps ranges of genome coordinates to chunk indexes, or a more specialised structure that supports overlap queries.

We then ran the af-dist calculation (Figs 3 and 4) on the VCF file using bcftools +af-dist as before. The elapsed time for this operation was 716.28 min CPU, 716.3 min elapsed. Repeating this operation for the same coordinates in Zarr (using Python code described in previous sections) gave a total CPU time of 2.32 min and elapsed time of 4.25 min. This is a 309X reduction in CPU burden and a 169X speed-up in elapsed time. It is worth noting here that bcftools +af-dist cannot be performed in parallel across multiple slices of a chromosome, and if we did want to run it on all of chromosome 2 we would need to concatenate the 106 VCF files. While af-dist itself is not a common operation, many tasks share this property of not being straightforwardly decomposable across multiple VCF files.

Finally, to illustrate performance on a common filtering task, we created a copy of the VCF chunk which contains only variants that pass some common filtering criteria using bcftools view -I -inciude “FORMAT/DP>10 & FORMAT/GQ>20”, following standard practices [e.g. 100, 96, 26]. This used 689.46 min CPU time, with an elapsed time of 689.48 min. In comparison, computing and storing a variant mask (i.e., a boolean value for each variant denoting whether it should be considered or not for analysis) based on the same criteria using Zarr consumed 1.96 min CPU time with an elapsed time of 11 min. This is a 358X reduction in CPU usage, and 63X reduction in elapsed time. There is an important distinction here between creating a copy of the data (an implicit part of VCF based workflows) and creating an additional *mask*. As Table 1 illustrates, call-level masks are cheap (the standard genotype missingness mask, call_genotype_mask, uses 0.49% of the overall storage) and variant or sample level masks require negligible storage. If downstream software can use configurable masks (at variant, sample and call level) rather than expecting full copies of the data, major storage savings and improvements in processing efficiency can be made. The transition from the manifold inefficiencies of present-day “copy-oriented” computing, to the “mask-oriented” analysis of large immutable, single-source datasets is a potentially transformational change enabled by Zarr.

## Discussion

VCF is a central element of modern genomics, facilitating the exchange of data in a large ecosystem of interoperating tools. Its current row-oriented form, however, is fundamentally inefficient, profoundly limiting the scalability of the present generation of bioinformatics tools. Large scale VCF data cannot currently be processed without incurring a substantial economic (and environmental [101]) cost. We have shown here that this is not a necessary situation, and that greatly improved efficiency can be achieved by using more appropriate storage representations tuned to the realities of modern computing. We have argued that Zarr provides a powerful basis for cloud-based storage and analysis of large-scale genetic variation data. We propose the VCF Zarr specification which losslessly maps VCF data to Zarr, and provide an efficient and scalable tool to perform conversion.

Zarr provides pragmatic solutions to some of the more pressing problems facing the analysis of large-scale genetic variation data, but it is not a panacea. Firstly, any dataset containing a variant with a large number of alleles (perhaps due to indels) will cause problems because the dimensions of fields are determined by their *maximum* dimension among all variants. In particular this is problematic for fields like PL in which the dimension depends quadratically on the number of alleles (although practical solutions have been suggested that we plan to implement [102]). Secondly, the design of VCF Zarr emphasises efficiency of analysis for a fixed dataset, and does not consider how samples (and the corresponding novel variants) should be added. Thirdly, Zarr works best for numerical data of a fixed dimension, and therefore may not suitable for representing the unstructured data often included in VCF INFO fields.

Nonetheless, there are numerous datasets that exist today that would likely reap significant benefits from being deployed in a cloud-native fashion using Zarr. Object stores typically allow for individual objects (chunks, in Zarr) to be associated with “tags”, which can then be used to associate storage class, user access control and encryption keys. Aside from the performance benefits we have focused on here provided by Zarr, the ability to (for example) use high-performance storage for commonly used data such as the variant position and more cost-effective storage classes for infrequently used bulk QC data should provide significant operational benefits. Granular access controls would similarly allow non-identifiable variant-level data to be shared relatively freely, with genotype and other data more tightly controlled as required. Even finer granularity is possible if samples are grouped by access level within chunks (padding partially filled chunks as needed and using an appropriate sample mask). Providing client applications direct access to the data over HTTP and delegating access control to the cloud provider makes custom web APIs [103] and cryptographic container formats [104] largely unnecessary in this setting.

The VCF Zarr specification and scalable vcf2zarr conversion utility provided here are a necessary starting point for such cloud-native biobank repositories and open up many possibilities, but significant investment and development would be needed to provide a viable alternative to standard bioinformatics workflows. Two initial directions for development, however, may quickly yield sufficient results to both greatly improve researcher productivity on large, centrally managed datasets such as Genomics England and motivate further research and development. The first direction is to provide compatibility with existing workflows via a “vcztools” command line utility which implements a subset of bcftools functionality (such as view and query) on a VCF Zarr dataset. Such a tool would speed up some common queries by orders of magnitude, and reduce the need for user orchestration of operations among manually split VCF chunks (large VCF datasets are typically split into hundreds of files; see the Genomics England case study). Datasets could then be hosted in cloud object stores, while still presenting file-like semantics for existing workflows. This could provide an evolutionary path, allowing established analysis workflows to co-exist with new Zarr-native approaches, working from the same primary data.

The second natural direction for development is to create these Zarr-native applications, which can take advantage of the efficient data representation across multiple programming languages (see Methods). The Python data science ecosystem, in particular, has a rich suite of powerful tools [e.g. 105, 92, 106, 93, 107] and is increasingly popular in recent biological applications [e.g. 108, 109, 110, 111]. Xarray [112] provides a unified interface for working with multi-dimensional arrays in Python, and libraries like Dask [113] and Cubed [114] allow these operations to be scaled out transparently across processors and clusters. This scaling is achieved by distributing calculations over grid-based array representations like Zarr, where chunks provide the basic unit for parallel computation. The VCF Zarr specification introduced here was created to facilitate work on a scalable genetics toolkit for Python [115] built on Xarray While the high-level facilities for distributed computation provided by Xarray are very powerful, they are not needed or indeed appropriate in all contexts. Our benchmarks here illustrate that working at the lowest level, by sequentially applying optimised kernels on a chunk-by-chunk basis is both straightforward to implement and highly performant. Thus, a range of possibilities exist in which developers can build utilities using the VCF Zarr specification using the appropriate level of abstraction and tool chain on a case-by-case basis.

While Zarr is now widely used across the sciences (see Methods) it was originally developed to store genetic variation data from the *Anopheles gambiae* 1000 Genomes Project [116] and is in active use in this setting [e.g. 117, 118]. The VCF Zarr specification presented here builds on this real-world experience but is still a draft proposal that would benefit from wider input across a range of applications. With some refinements and sufficient uptake it may be suitable for standardisation [2]. The benefits of Zarr are substantial, and, in certain settings, worth the cost of retooling away from classical file-oriented workflows. For example, the MalariaGEN Vector Observatory currently uses Zarr to store data from whole-genome sequencing of 23,000 *Anopheles* mosquitoes from 31 African countries [119]. The data is hosted in Google Cloud Storage and can be analysed interactively using free cloud computing services like Google Colab, enabling the use of data by scientists in malaria-endemic countries where access to local computing infrastructure and sufficient network bandwidth to download large datasets may be limited. VCF Zarr could similarly reduce the costs of analysing large-scale human data, and effectively open access to biobanks for a much broader group of researchers than currently possible.

## Methods

### Zarr and block-based compression

In the interest of completeness it is useful to provide a high-level overview of Zarr and the technologies that it depends upon. Zarr is a specialised format for storing large-scale *n*-dimensional data (arrays). Arrays are split into chunks, which are compressed and stored separately. Chunks are addressed by their indexes along the dimensions of the array, and the compressed data associated with this key. Chunks can be stored in individual files (as we do here), but a wide array of different storage backends are supported including cloud object stores and NoSQL databases; in principle, Zarr can store data in any key-value store. Metadata describing the array and its properties is then stored in JSON format along with the chunks. The simplicity and transparency of this design has substantial advantages over other technologies such as HDF5 [120] which are relatively complex and opaque. This simplicity has led to numerous implementations of the Zarr specification being developed, ranging from the mature Zarr-Python [121] and TensorStore [122] implementations to more experimental extensions to packages like GDAL [123], NetCDF [124], N5 [125] and xtensor [126] as well as standalone libraries for JavaScript [127], Julia [128], Rust [129] and R [130].

Zarr is flexible in allowing different compression codecs and pre-compression filters to be specified on a per-array basis. Two key technologies often used in conjunction with Zarr are the Blosc meta-compressor [89] and Zstandard compression algorithm [88]. Blosc is a high-performance compressor optimised for numerical data which uses “blocking” [89] to optimise CPU-cache access patterns, as well as highly optimised bit and byte shuffle filters. Remarkably, on highly compressible datasets, Blosc decompression can be faster than memcpy. Blosc is written in C, with APIs for C, Python, Julia, Rust and others. Blosc is a “meta-compressor” because it provides access to several different compression codecs. The Zstandard codec is of particular interest here as it achieves very high compression ratios with good decompression speeds (Figs S1, S3). Zstandard is also used in several recent VCF compression methods [e.g. 57, 58].

Scientific datasets are increasingly overwhelming the classical model of downloading and analysing locally, and are migrating to centralised cloud repositories [36, 85]. The combination of Zarr’s simple and cloud-friendly storage of data chunks with state-of-the-art compression methods has led to Zarr gaining significant traction in these settings. Multiple petabyte-scale datasets are now stored using Zarr [e.g. 86, 131, 132] or under active consideration for migration [84, 133]. The Open GeoSpatial consortium has formally recognised Zarr as a community standard [134] and has formed a new GeoZarr Standards Working Group to establish a Zarr encoding for geospatial data [135].

Zarr has recently been gaining popularity in biological applications. The Open Microscopy Environment has developed OME-Zarr [136] as one of its “next generation” cloud ready file formats [85]. OME-Zarr already has a rich suite of supporting tools [136, 137]. Zarr has also seen recent uptake in single-cell single-cell genomics [138, 139] and multimodal spatial omics data [140, 141]. Recent additions using Zarr include the application of deep learning models to genomic sequence data [142], storage and manipulation of large-scale linkage disequilibrium matrices [143], and a browser for genetic variation data [144].

### The VCF Zarr specification

The VCF Zarr specification is a direct mapping from the VCF data model to a chunked binary array format using Zarr, and is an evolution of the Zarr format used in the scikit-allel package [145]. VCF Zarr takes advantage of Zarr’s hierarchical structure by representing a VCF file as a top-level Zarr group containing Zarr arrays. Each VCF field (fixed fields, INFO fields, and FORMAT fields) is represented as a separate array in the Zarr hierarchy Some of the structures from the VCF header are also represented as arrays, including contigs, filters, and samples.

The specification defines the name, shape, dimension names, and data type for each array in the Zarr store. These “logical” properties are mandated, in contrast to “physical” Zarr array properties such as chunk sizes and compression, which can be freely chosen by the implementation. This separation makes it straightforward for tools and applications to consume VCF Zarr data since the data has a well-defined structure, while allowing implementations enough room to optimise chunk sizes and compression according to the application’s needs.

The specification defines a clear mapping of VCF field names (keys) to array names, VCF Number to array shape, and VCF Type to array data type. To take one example, consider the VCF AD genotype field defined by the following VCF header: ##FORMAT=<ID=AD,Number=A,Type=Integer,Description=“Allele Depths”>. The FORMAT key ID maps to an array name of call_AD (FORMAT fields have a call_ prefix, while INFO fields have a variant_ prefix; both are followed by the key name). Arrays corresponding to FORMAT fields are 3-dimensional with shapes that look like (variants, samples, <Number>) in general. In this case, the Number A entry indicates that the field has one value per alternate allele, which in VCF Zarr is represented as the alt_alleles dimension name, so the shape of this array is (variants, samples, alt_alleles). The VCF Integer type can be represented as any Zarr integer type, and the specification doesn’t mandate particular integer widths. The vcf2zarr (see the next section) conversion utility chooses the narrowest integer width that can represent the data in each field.

An important aspect of VCF Zarr is that field dimensions are global and fixed, and defined as the maximum across all rows. Continuing the example above, the third dimension of the array is the maximum number of alternate alleles across *all* variants. For variants at which there are less than the maximum number of alternative alleles, the third dimension of the call_AD array is padded with a sentinel value (−2 for integers and a specific non-signalling NaN for floats). While this is not a problem in practice for datasets in which all four bases are observed, it is a substantial issue for fields that have a quadratic dependency on the number of alleles (Number=G) such as PL. Such fields are already known to cause significant problems, and the “local alleles” proposal provides an elegant solution [102]. As this approach is on a likely path to standardisation [146], we plan to include support in later versions of VCF Zarr.

The VCF Zarr specification can represent anything described by BCF (which is somewhat more restrictive than VCF) except for two corner cases related to the encoding of missing data. Firstly, VCF Zarr does not distinguish between a field that is not present and one that is present but contains missing data. For example, a variant with an INFO field NS=. is represented in the same way in VCF Zarr as an INFO field with no NS key. Secondly, because of the use of sentinel values to represent missing and fill values for integers (−1 and −2, respectively), a field containing these original values cannot be stored. In practice this doesn’t seem to be much of an issue (we have not found a real VCF that contains negative integers). However, if −1 and −2 need to be stored, a float field can be used without issues.

The VCF Zarr specification is general and can be mapped to file formats such as PLINK [15, 16] and BGEN [17] with some minor extensions.

### vcf2zarr

Converting VCF to Zarr at Biobank scale is challenging. One problem is to determine the dimension of fields, (i.e., finding the maximum number of alternate alleles and the maximum size of Number=. fields) which requires a full pass through the data. Another challenge is to keep memory usage within reasonable limits: although we can view each record in the VCF one-by-one, we must buffer a full chunk (10,000 variants is the default in vcf2zarr) in the variants dimension for each of the fields to convert to Zarr. For VCFs with many FORMAT fields and large numbers of samples this can require tens of gigabytes of RAM per worker, making parallelism difficult. Reading the VCF multiple times for different fields is possible, but would be prohibitively slow for multi-terabyte VCFs.

The vcf2zarr utility solves this problem by first converting the VCF data (which can be split across many files) into an Intermediate Columnar Format (ICF). The vcf2zarr explode command takes a set of VCFs, and reads through them using cyvcf2 [147], storing each field independently in (approximately) fixed-size compressed chunks. Large files can be partitioned based on information extracted from the CSI or Tabix indexes, and so different parts of a file can be converted to ICF in parallel. Once all partitions have completed, information about the number of records in each partition and chunk of a given field is stored so that the record at a particular index can be efficiently retrieved. Summaries such as maximum dimension and the minimum and maximum value of each field are also maintained, to aid choice of data types later. A set of VCF files can be converted to intermediate columnar format in parallel on a single machine using the explode command, or can be distributed across a cluster using the dexplode-init, dexplode-partition and dexplode-finalise commands.

Once the VCF data has been converted to the intermediate columnar format, it can then be converted to Zarr using the vcf2zarr encode command. By default we choose integer widths based on the maximum and minimum values observed during conversion to ICF along with reasonable compressor defaults (see next section). Default choices can be modified by generating a JSON-formatted storage schema, which can be edited and supplied as an argument to encode. Encoding a given field (for example, call_AD) involves creating a buffer to hold a full variant-chunk of the array in question, and then sequentially filling this buffer with values read from ICF and flushing to file. Similar to the explode command, encoding to Zarr can be done in parallel on a single machine using the encode command, or can be distributed across a cluster using the dencode-init, dencode-partition and dencode-finalise commands. The distributed commands are fault-tolerant, reporting any failed partitions so that they can be retried.

### Choosing default compressor settings

To inform the choice of compression settings across different fields in VCF data, we analysed their effect on compression ratio on recent high-coverage WGS data from the 1000 Genomes project [148]. We began by downloading the first 100,000 lines of the VCF for chromosome 22 (giving a 1.1GiB compressed VCF) and converted to Zarr using vcf2zarr with default settings. We then systematically examined the effects of varying chunk sizes and compressor settings on the compression ratio for call-level fields. We excluded call_PL from this analysis as it requires conversion to a “local alleles” encoding [102] to be efficient, which is planned for implementation in a future version of vcf2zarr.

Fig S3 shows the effect of varying compression codecs in Blosc. The combination of outstanding compression performance and competitive decoding speed (Fig S1) makes zstd a good default choice.

The shuffle parameter in the Blosc meta-compressor [89] can result in substantially better compression, albeit at the cost of somewhat slower decoding (see Fig S1). Fig S4 shows the effect of bit shuffle (grouping together bits at the same position across bytes before compression), and byte shuffle (grouping together bytes at the sample position across words before compression) on compression ratio. Bit shuffle provides a significant improvement in compression for the call_genotype field because the vast majority of genotype calls will be 0 or 1, and therefore bits 1 to 7 will be 0. Thus, grouping these bits together will lead to significantly better compression. This strategy also works well when compressing boolean fields stored as 8 bit integers, where the top 7 bits are always 0. In practice, boolean fields stored in this way have very similar compression to using a bit-packing pre-compression filter (data not shown). Although byte shuffle leads to somewhat better compression for call_AD and call_DP, it gives substantially worse compression on call_AB than no shuffling. The default in vcf2zarr is therefore to use bit shuffle for call_genotype and all boolean fields, and to not use byte shuffling on any field. These defaults can be easily overruled, however, by outputting and modifying a JSON formatted storage schema before encoding to Zarr.

Fig S5 shows that chunk size has a weak influence on compression ratio for most fields. Increasing sample chunk size slightly increases compression on call_AB, and has no effect on less compressible fields. Variant chunk size appears to have almost no effect on compression ratio. Interestingly, the choice of chunk size along the sample dimension for the genotype matrix does have a significant effect. With six evenly spaced points between 100 and 2504, Fig S5A shows a somewhat unpredictable relationship between sample chunk size and compression ratio. The more fine-grained analysis of Fig S6 shows that three distinct trend lines emerge depending on the chunk size divisibility, with the modulus (i.e., the remainder in the last chunk) also having a minor effect. At greater than 40X, compression ratio is high in all cases, and given that genotypes contribute relatively little to the total storage of real datasets (Table 1) the effect will likely be fairly minor in practice. Thus, we do not expect the choice of chunk size to have a significant impact on overall storage usage, and so choice maybe determined by other considerations such as expected data access patterns.

### Benchmarks

In this section we describe the methodology used for the simulation-based benchmarks of Figs 2,3, 4 and 5. The benchmarks use data simulated by conditioning on a large pedigree of French-Canadians using msprime [149], which have been shown to follow patterns observed in real data from the same population to a remarkable degree [87]. We begin by downloading the simulated ancestral recombination graph [150, 151, 152] for chromosome 21 from Zenodo [153] in compressed tszip format. This 552M file contains the simulated ancestry and mutations for 1.4 million present-day samples. We then subset the full simulation down to 10^1^, 10^2^, …, 10^6^ samples using ARG simplification [154, 152], storing the subsets in tskit format [155]. Note that this procedure captures the growth in the number of variants (shown in the top x-axis labels) as we increase sample sizes as a natural consequence of population-genetic processes. As a result of simulated mutational processes, most sites have one alternate allele, with 7.9% having two and 0.2% having three alternate alleles in the 10^6^ samples dataset. We then export the variation data from each subset to VCF using tskit vcf subset.ts | bgzip > subset.vcf.gz as the starting point for other tools.

We used bcftools version 1.18, Savvy 2.1.0, Genozip 5.0.26, vcf2zarr 0.0.9, and Zarr-Python 2.17.2. All tools used default settings, unless otherwise stated. All simulation-based benchmarks were performed on a dual CPU (Intel Xeon E5–2680 v2) server with 256GiB of RAM running Debian GNU/Linux 11. To ensure that the true effects of having data distributed over a large number of files were reported, benchmarks for Zarr and Savvy were performed on a cold disk cache by running echo 3 | sudo tee /proc/sys/vm/drop_caches before each run. The I/O subsystem used is based on a RAID 5 of 12 SATA hard drives. For the CPU time benchmarks we measure the sum of the total user and system times required to execute the full command (as reported by GNU time) as well as elapsed wall-clock time. Total CPU time is shown as a solid line, with wall-clock time as a dashed line of the same colour. In the case of pipelines, where some processing is conducted concurrently wall-clock time can be less than total CPU (e.g. genozip in Fig 3). When I/0 costs are significant, wall-clock time can be greater than total CPU (e.g. Zarr and Savvy in Fig 4). Each tool was instructed to use one thread, where the options were provided. Where possible in pipelines we use uncompressed BCF output (−Ou) to make processing more efficient [146]. We do not use BCF output in genozip because it is not supported directly.

Because bcftools +af-dist requires the AF INFO field and this is not kept in sync by bcftools view (although the AC and AN fields are), the subset calculation for Fig 4 requires an additional step. The resulting pipeline is bcftools view -r REGION -S SAMPLESFILE -IOu BCFFILE | bcftools +fill-tags -Ou | bcftools +af-dist. Genozip similarly requires a +fill-tags step in the pipeline.

## Supplementary Material

1

## Figures and Tables

**Table 1.** Summary for a selection of the largest VCF Zarr columns produced for Genomics England aggV2 VCFs on chromosome 2 using vcf2zarr default settings. Each field is stored independently as a Zarr array with the given type (sufficient to represent all values in the data). We show the total storage consumed (reported via du) in power-of-two units, and the compression ratio achieved on that array. We also show the percentage of the overall storage that each array consumes (omitting values < 0.01%).

Field	type	storage	compress	%total
/call_AD	int16	658.4G	26	25.35%
/call_GQ	int16	654.5G	13	25.20%
/call_DP	int16	570.0G	15	21.95%
/call_DPF	int16	447.1G	20	17.22%
/call_PL	int16	162.6G	160	6.26%
/call_GQX	int16	41.0G	210	1.58%
/call_FT	string	25.0G	1400	0.96%
/call_genotype	int8	21.5G	410	0.83%
/call_genotype_mask	bool	12.8G	680	0.49%
/call_genotype_phased	bool	2.4G	1900	0.09%
/call_PS	int8	383.4M	12000	0.01%
/variant_position	int32	111.6M	2	
/variant_quality	float32	87.4M	2.6	
/variant_allele	string	69.3M	13	
/variant_AN	int32	47.3M	4.8	
/variant_filter	bool	6.4M	570	
/sample_id	str	268.1K	2.3	

## Key Points

- VCF is widely supported, and the underlying data model entrenched in bioinformatics pipelines.
- The standard row-wise encoding as text (or binary) is inherently inefficient for large-scale data processing.
- The Zarr format provides an efficient solution, by encoding fields in the VCF separately in chunk-compressed binary format.

## List of abbreviations

ICF: Intermediate Columnar Format
GWAS: Genome Wide Association Study
PBWT: Positional Burrows-Wheeler Transform
QC: Quality Control
UKB: UK Biobank
VCF: Variant Call Format
WGS: Whole Genome Sequence
