# Supplementary material for: Analysis-ready VCF at Biobank scale using Zarr
Source: bioRxiv. 2025 Feb 6:2024.06.11.598241. Originally published 2024 Jun 12. Preprint. [Version 3] doi: 10.1101/2024.06.11.598241 (PMC11195102; doi:10.1101/2024.06.11.598241)
Supplement: Supplement 1 [file NIHPP2024.06.11.598241v3-supplement-1.pdf]

bioRxiv preprint doi: https://doi.org/10.1101/2024.06.11.598241; this version posted February 6, 2025. The copyright holder for this preprint (which was not certified by peer review) is the author/funder, who has granted bioRxiv a license to display the preprint in perpetuity. It is made available under aCC-BY 4.0 International license.

Supplementary Material

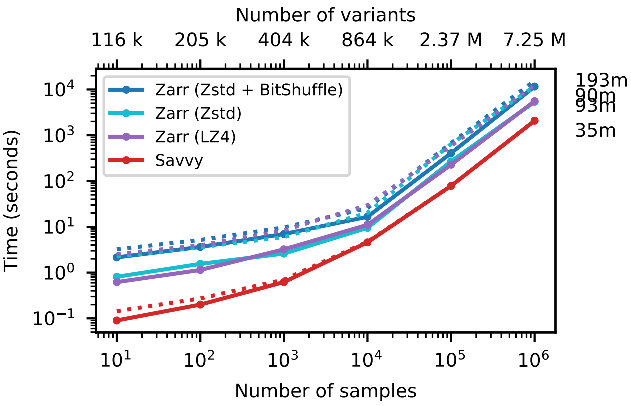

Figure S1. Genotype decoding performance. Total CPU time required to decode genotypes into memory using the Zarr-Python and Savvy C++ APIs for the data in Figure 2. Elapsed time is also reported (dotted line). This corresponds to a maximum rate of 1.2 GiB/s for Zarr (Zstd + BitShuffle), 2.7 GiB/s Zarr (Zstd), 2.9 GiB/s Zarr (LZ4), and 6.6 GiB/s for Savvy.

bioRxiv preprint doi: https://doi.org/10.1101/2024.06.11.598241; this version posted February 6, 2025. The copyright holder for this preprint (which was not certified by peer review) is the author/funder, who has granted bioRxiv a license to display the preprint in perpetuity. It is made available under aCC-BY 4.0 International license.

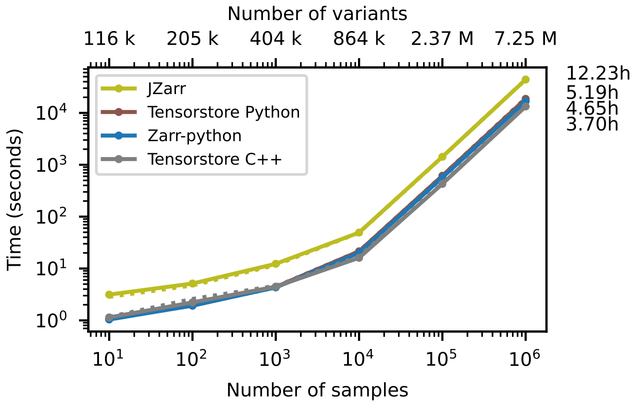

Figure S2. Whole-matrix computation performance using different Zarr implementations. Total CPU time required to run the af-dist calculation the data in Figure 2 using different Zarr implementations. Elapsed time is also reported (dotted line). These benchmarks were run on an 8-core CPU (Intel i7-9700) with 32 GiB RAM running Linux Mint 21.3 with data on an NVMe SSD. Note the difference between the time reported here and in Fig 3 for Zarr-Python is due to different hardware platforms.

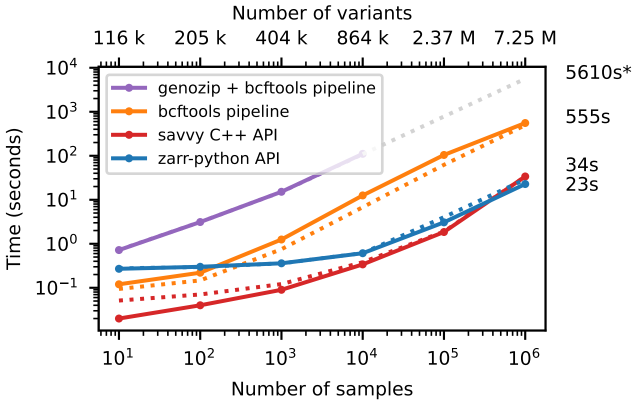

Figure S3. Compute performance on a large subset of the genotype matrix. Total CPU time required to run the af-dist calculation for a subset of half of the samples and 10000 variants from the middle of the matrix for the data in Figure 2. Elapsed time is also reported (dotted line). Genozip did not run for $n > 10^4$ samples because it does not support a file to specify sample IDs, and the command line was therefore too long for the shell to execute.

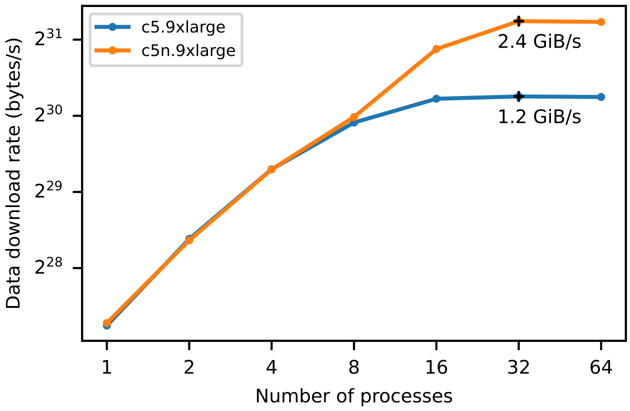

Figure S5. Chunk data download rate on AWS. Using the prototype parallel Zarr implementation (see Methods) we measured the rate at which chunks of compressed genotype data can be downloaded on two different instance types.

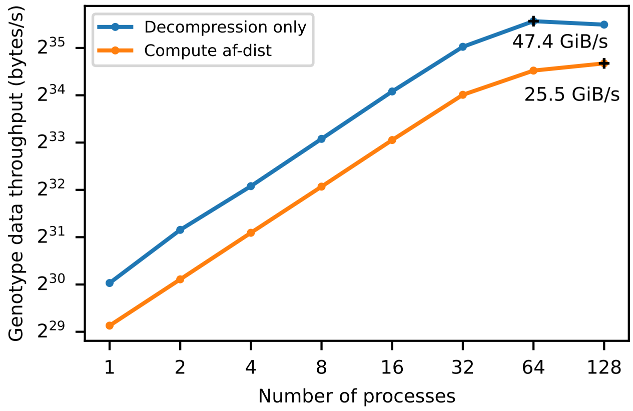

Figure S4. Scalability of genotype data processing on AWS. Using the prototype parallel Zarr implementation (see Methods) we measured the rate genotype data can be decoded to memory (decompression only) and the rate at which we can perform the full af-dist calculation as we vary the number of parallel processes.

bioRxiv preprint doi: https://doi.org/10.1101/2024.06.11.598241; this version posted February 6, 2025. The copyright holder for this preprint (which was not certified by peer review) is the author/funder, who has granted bioRxiv a license to display the preprint in perpetuity. It is made available under aCC-BY 4.0 International license.

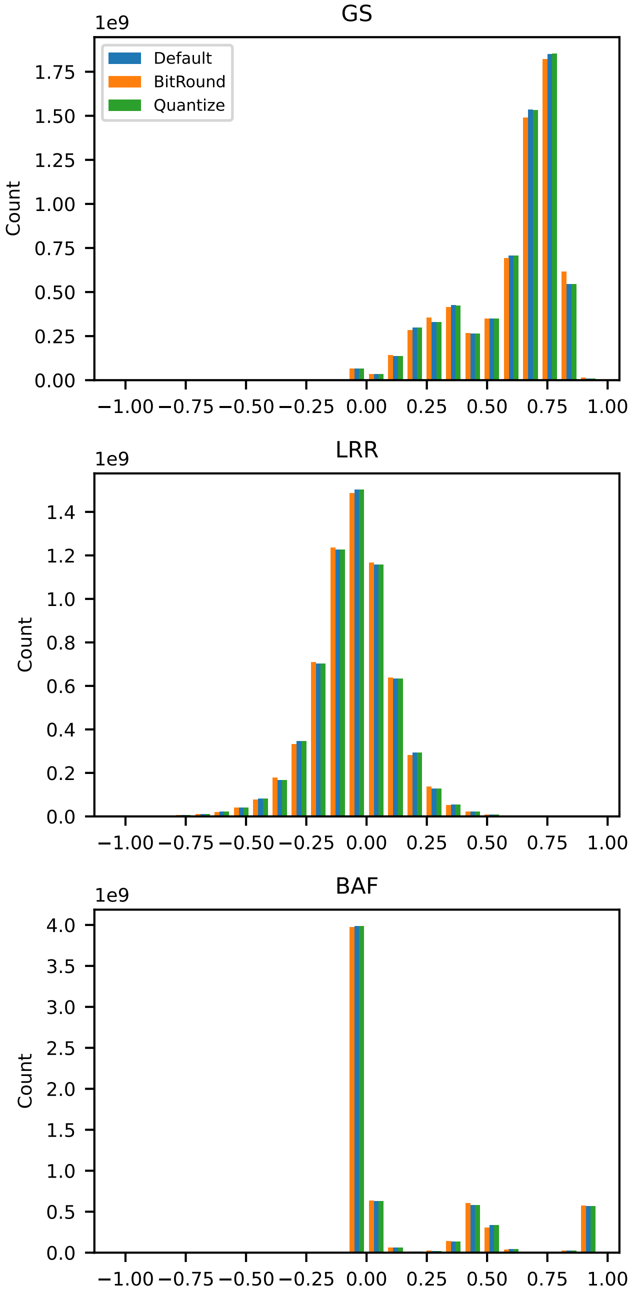

Figure S6. Distribution of values in the GS, LRR and BAF fields in the Our Future Health data with no truncation (Default), and truncation using the BitRound and Quantize filters.

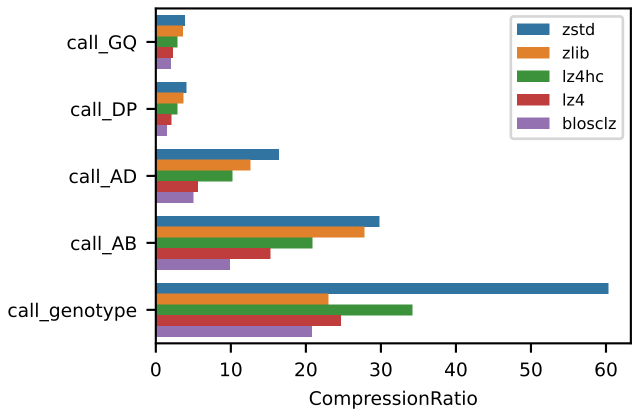

Figure S7. Effects of Blosc compression codec on compression ratio on call-level fields in 1000 Genomes data. In all cases compression level=7 was used, with a variant chunk size of 10,000 and sample chunk size of 1,000. Bit shuffle was used for call_genotype, and no shuffle used for the other fields.

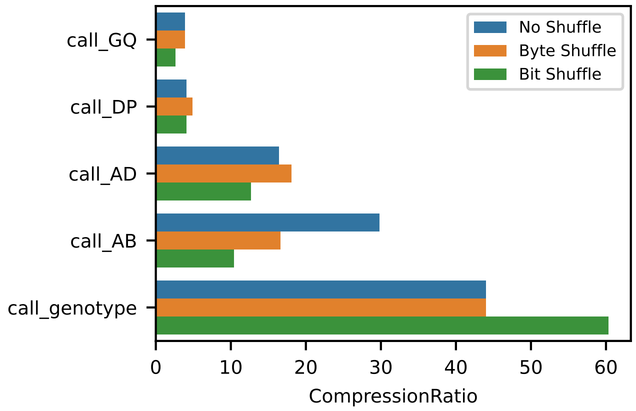

Figure S8. Effects of Blosc shuffle settings on compression ratio on call-level fields in 1000 Genomes data. In all cases the zstd compressor with compression level=7 was used, with a variant chunk size of 10,000 and sample chunk size of 1,000.

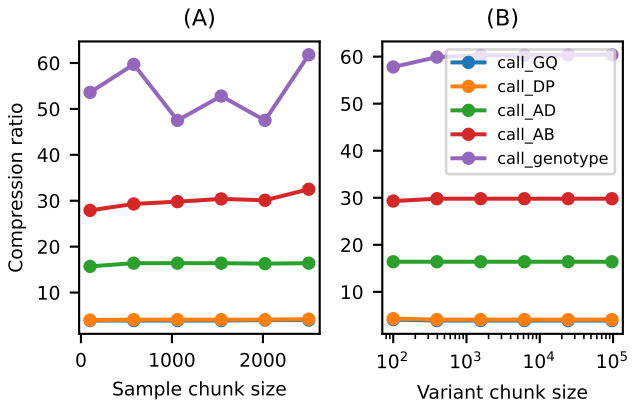

Figure S9. Effects of chunk sizes on compression ratio on call-level fields in 1000 Genomes data. (A) Varying sample chunk size, holding variant chunk size fixed at 10,000. (B) Varying variant chunk size, holding sample chunk size fixed at 1,000. In all cases the zstd compressor with compression level=7 was used. Bit shuffle was used for call_genotype, and no shuffle used for the other fields. Values are chosen to be evenly spaced on a linear scale between 100 and 2504 (the number of samples) in (A) and evenly spaced between 100 and 96514 on a log scale in (B).

bioRxiv preprint doi: https://doi.org/10.1101/2024.06.11.598241; this version posted February 6, 2025. The copyright holder for this preprint (which was not certified by peer review) is the author/funder, who has granted bioRxiv a license to display the preprint in perpetuity. It is made available under aCC-BY 4.0 International license.

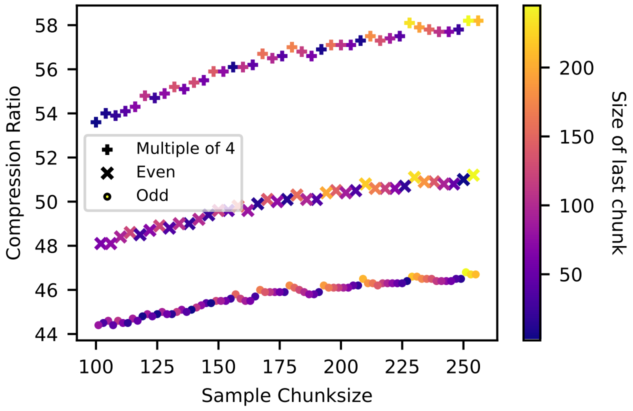

Figure S10. Effects of sample chunk size on compression ratio on the call_genotype field in 1000 Genomes data. The same analysis as in Fig S9, except we only consider call_genotype and we examine all sample chunk sizes from 100 to 256. Distinct trend-lines emerge for odd, even and multiple-of-four chunk sizes (shown by markers). The size of the final chunk also has a minor effect (shown by colour).
